# Genetic and Molecular Regulations of Neuronal Activity

**DOI:** 10.3390/ijms242216191

**Published:** 2023-11-10

**Authors:** Marcella Reale, Cesar Borlongan, Anna R. Carta, Nigel H. Greig

**Affiliations:** 1 Department of Innovative Technologies in Medicine and Dentistry, Unit of Immunodiagnostic and Molecular Pathology, University “G.d’Annunzio”, 66100 Chieti, Italy; 2 Department of Neurosurgery, University of South Florida College Medicine, MDC 78, 12901 Bruce B Downs Blvd., Tampa, FL 33612, USA; cborlong@usf.edu; 3 Department of Biomedical Sciences, University of Cagliari, 09124 Cagliari, Italy; acarta@unica.it; 4 Translational Gerontology Branch, Intramural Research Program, National Institute on Aging, National Institutes of Health, Baltimore, MD 21224, USA; greign@grc.nia.nih.gov

This Special Issue of the *International Journal of Molecular Sciences* (*IJMS*) focuses on ‘Genetic and Molecular Regulations of Neuronal Activity’. Constant advances in molecular biology and gene regulation knowledge continue to drive innovations in neuroscience. In this regard, the regulation of the transcription of many genes, together with the splicing, trafficking, and translation of mRNA, is intricately involved in controlling protein levels and their ultimate cellular localization and role. Within neurons, the interaction of multiple gene regulatory mechanisms is essential to healthy development, maintenance through life, and function of the nervous system. Genetic mutations and loss of regulation of gene expression undoubtedly play a critical role in aberrant neuronal development and the occurrence of neurological disorders. Dissecting the regulatory pathways provides us insight into potential mechanisms underpinning neurodevelopment as well as neuropsychiatric and neurodegenerative disorders and potentially opens new avenues through which future treatments may arise.

In this light, this Special Issue adds five manuscripts to the scientific literature to deepen our understanding of how neuronal activity is optimized and maintained. A primary paper by Marina Kopaeva et al. [1] evaluates the role of the globular multifunctional glycoprotein (i.e., lactoferrin) in regulating neuronal processes that augment long-term neuroplasticity. Interestingly, lactoferrin is a member of the transferrin protein family that supports the transfer of iron into cells and controls free iron levels in blood and biological fluids. Lactoferrin exists in different polymeric forms (monomers to tetramers), is present in most exocrine secretions (tears, saliva, milk, etc.), and, additionally, is a member of the innate immune system with described anti-bacterial, anti-viral, anti-inflammatory, anti-allergic, anti-cancer, anti-parasitic, and catalytic properties [2]. Similar to numerous other proteins and peptides, lactoferrin’s role can vary depending on its location and microenvironment, and this appears to be the case in the brain. The presence of the lactoferrin receptor on brain capillary endothelial cells and neurons allows the glycoprotein to cross the blood-brain barrier and enter neurons, and it appears to impact a wide range of neuronal processes by modulating the expression of genes involved in long-term neuroplasticity and neurological disorders (including BDNF, CREB, CAMK, and others) [3]. The article by Kopaeva et al. [1] provides insight into how neurological processes associated with lactoferrin are mediated in relation to its cellular localization and actions on the iconic transcription factor c-Fos in primary neuronal cultures.

The primary paper by Nicla Romano et al. [4] defines the role of ribosomal Receptor for Active C Kinase 1 (RACK1) in regulating neuronal cell dendritic arborization by suppressing Fragile X Mental Retardation 1 Protein (FMRP) activity, thereby providing a new mechanism underlying how FMRP activity can contribute to altered neurodevelopment, as evident in Fragile X Syndrome (FXS). FXS, an X-linked dominant condition, is a leading cause of inherited intellectual disability and is predominantly caused by the presence of a triplet repeat expansion in the 5′ untranslated region of the *FMR1* gene. This leads to DNA methylation, transcriptional inactivation, and the loss of the gene product, FMRP. One of the key roles of FMRP is to regulate mRNA metabolism in the brain and, thereby, to control the expression of key molecules involved in receptor signaling and spine morphology [5]. In large part, FMRP fulfills this role by being an RNA-binding protein that shuttles between the nucleus and cytoplasm, regulating the stability, subcellular transport, and translation of target neuronal mRNAs encoding for proteins involved in synaptic structure and function [6]. A loss or absence of FMRP thus leads to increased protein synthesis. Additionally, FMRP appears vital in neural stem and progenitor cell proliferation, differentiation, and survival, implying further processes that may underpin common facets of neurodevelopmental disorders. RACK1 also appears to function as a ribosomal binding protein [7], and Romano et al. [4] demonstrate that its binding with FMRP can modulate the latter’s actions.

The review article by Riccardo Zocchi et al. [8] deciphers the numerous elements that, together, determine the structural conformation of microtubules that provide a vital element of the cytoskeleton of all eukaryotic cells. The focus of Zocchi et al. [8] on the nervous system and the ‘tubulin code’ illuminates how the molecular determinants of microtubules can optimize and impact microtubule function and behavior across neuronal events, such as neuron migration, maturation, and axonal transport. Microtubules comprise assemblies of α- and β-tubulin heterodimers, and although these are evolutionarily highly conserved, the resulting filaments they generate can adapt to a huge variety of functions. In this regard, microtubules are considered to be intrinsically dynamic, being able to alternate between phases of polymerization and impromptu depolymerization, involving a practice termed ‘dynamic instability’. Furthermore, rather than function alone, microtubules interact with a broad collection of microtubule-associated proteins (MAPs) that manipulate their fabrication, structure, dynamics, and stability [9]. Nine α-tubulin and nine β-tubulin genes have been characterized in mammals, which allow the expression and incorporation of different tubulin isotypes into the microtubule framework, and these, together with the post-translational modification of tubulins, can functionally modulate microtubules and provide the ‘tubulin code’ [10] to allow fine regulation of function and adaption to specific and/or changing tasks of neuronal cells during development, aging, health, and disease [11].

The review article by Maria Ricci et al. [12] provides a detailed and up-to-date synopsis of new molecular and neuroimaging approaches to aid the diagnosis of fibromyalgia. Fibromyalgia is a disorder distinguished by chronic musculoskeletal pain. Its key symptoms are muscle stiffness, joint stiffness, fatigue, insomnia, mood disorders that commonly include anxiety and/or depression, cognitive impairment, a general sensitivity, and an incapacity to accomplish normal daily activities. Fibromyalgia is often accompanied by other conditions, including infections, rheumatic diseases, diabetes, and neurological and neuropsychiatric disorders, with stress or psychological trauma potentially being a precipitating factor. There appears to be a genetic component associated with susceptibility, with reported polymorphisms in a variety of genes associated with the serotoninergic, dopaminergic, and catecholaminergic systems [13]. Additionally, a neuroinflammatory component has been described [14]. However, although fibromyalgia is estimated to affect between 2% and 4% of the world’s population, its cause and pathophysiology remain largely unknown, and the current diagnosis is therefore almost exclusively based on clinical assessment. In this light, the evaluation of the role of molecular and functional imaging [12] could substantially contribute to the diagnosis of fibromyalgia, responses to therapy, and identification of mechanisms driving the disorder.

Finally, the review by Yelizhati Ruzha et al. [15] provides the current status of knowledge on the role of the glycoprotein vitronectin and its receptors (i.e., integrins) within the nervous system, their association with neurodegenerative disorders, and current drugs targeted towards them. A key feature of the nervous system, particularly during its development but also in the adult brain following an injury, is the remarkable capacity of neurons to extend axons over lengthy distances and selectively navigate them to precise destinations to generate synapses and functional, critical networks. The precise creation of correct synaptic structures and neuronal connections is vital to supporting healthy brain development and an optimally functional adult brain [16]. In this regard, the integrin family of cell adhesion receptors, together with their ligands (i.e., extracellular matrix glycoproteins that include vitronectin), perform indispensable roles in the processes that control neuronal connectivity. This encompasses neurite outgrowth, the establishment and maintenance of synapses, and synaptic plasticity to adapt and optimize the nervous system to changing physiological needs. The complexity of integrin–vitronectin interactions has been comprehensively studied in non-neuronal tissues [17]. However, although widely expressed in the brain, their characterization and multifaceted neuronal roles have been less studied. Furthermore, integrin crosstalk with other receptor systems appears to be a recurrent regulatory mechanism to achieve and maintain optimal neuronal function and opens numerous corridors to support pharmacological modulation. In this light, the review by Ruzha et al. [15] is a valuable addition to the scientific literature. 

In closure, we end this editorial by thanking all article authors who enthusiastically responded to our request by contributing to this *IJMS* Special Issue. We, moreover, extend our huge appreciation to the peer reviewers for the time and expertise that each altruistically contributed by evaluating and suggesting appropriate revisions to the Special Issue articles to support their acceptance and publication. Our hope, as the Special Issue Editors, is that the published articles will both expertly inform the scientific community and challenge it to contemplate and probe for answers to the many questions that remain on the underpinnings of genetic and molecular regulations of neuronal activity and, thereby, help move scientific discovery towards improving public health.
